# What should medical students know about artificial intelligence in medicine?

**DOI:** 10.3352/jeehp.2019.16.18

**Published:** 2019-07-03

**Authors:** Seong Ho Park, Kyung-Hyun Do, Sungwon Kim, Joo Hyun Park, Young-Suk Lim

**Affiliations:** 1 Department of Radiology and Research Institute of Radiology, Asan Medical Center, University of Ulsan College of Medicine, Seoul, Korea; 2 Department of Radiology, Research Institute of Radiological Science and Center for Clinical Image Data Science, Severance Hospital, Yonsei University College of Medicine, Seoul, Korea; 3 Department of Medical Education, University of Ulsan College of Medicine, Seoul, Korea; 4 Department of Gastroenterology, Asan Medical Center, University of Ulsan College of Medicine, Seoul, Korea; The Catholic University of Korea, Korea

**Keywords:** Artificial intelligence, Machine learning, Deep learning, Medical students

## Abstract

Artificial intelligence (AI) is expected to affect various fields of medicine substantially and has the potential to improve many aspects of healthcare. However, AI has been creating much hype, too. In applying AI technology to patients, medical professionals should be able to resolve any anxiety, confusion, and questions that patients and the public may have. Also, they are responsible for ensuring that AI becomes a technology beneficial for patient care. These make the acquisition of sound knowledge and experience about AI a task of high importance for medical students. Preparing for AI does not merely mean learning information technology such as computer programming. One should acquire sufficient knowledge of basic and clinical medicines, data science, biostatistics, and evidence-based medicine. As a medical student, one should not passively accept stories related to AI in medicine in the media and on the Internet. Medical students should try to develop abilities to distinguish correct information from hype and spin and even capabilities to create thoroughly validated, trustworthy information for patients and the public.

## Introduction

The use of artificial intelligence (AI) for medicine has recently drawn much attention due to the advances in machine learning techniques involving multiple layers of artificial neural networks trained on big data, i.e., deep learning [1,2]. AI is expected to affect various fields of medicine substantially and has the potential to improve many aspects of healthcare [1]. On the downside, AI has been creating much hype, too. It is not difficult to find on the Internet stories about how fast and accurately modern AI software programs can analyze the patient’s medical information and automatically present diagnoses, even more precisely than human experts, with a nuance that AI will soon dominate the medical practice. However, these stories are mostly quite exaggerated or, at best, explain the matter only superficially. In reality, few AI techniques are currently used in medical practice.

Nevertheless, at the time when the current medical students will commence their career as medical professionals after completion of studies and training, various AI software tools will likely be used in clinical practice. In applying AI technology to patients, medical professionals are not ones who are in the backseat but should be in the driver’s seat. They should be able to resolve any anxiety, confusion, and questions that patients and the public may have about applying AI to medicine. Medical professionals are also responsible for ensuring that AI becomes a technology beneficial for patient care. These make the acquisition of sound knowledge and experience about AI a task of high importance for medical students. The purpose of this article is to provide a succinct summary of the current state of AI from a medical viewpoint and suggest what medical students should do to prepare for the era of AI in medicine.

## Artificial intelligence-related terms

**Fig. 1.**
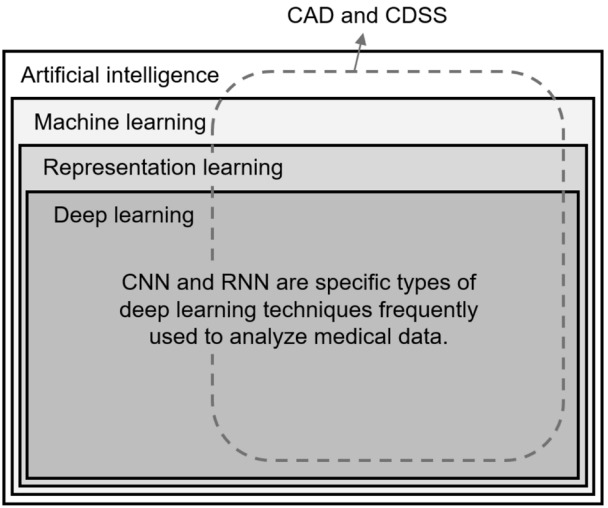
Hierarchy of artificial intelligence-related terms. CAD and CDSS are the most common types of software tools in the application of AI in medicine. CAD, computer-aided detection/diagnosis; CDSS, clinical decision support system; CNN, convolutional neural network; RNN, recurrent neural network.

AI is a broad term that refers to algorithms that allow computers to perform tasks requiring human cognitive abilities. With the recent development of deep learning technology, the terms ‘deep learning’ and ‘AI’ are increasingly often used as synonyms. The hierarchy of common AI-related terms are visually summarized in Fig. 1 [3].

## Hype versus reality

Since the Go champion Sedol Lee was defeated in early 2016 by Google’s AlphaGo developed using deep learning technologies, AI has often been referred to by lay media and some people as a technology that would replace many physicians in the foreseeable future. However, 3 years later, now ironically, worries exist that many companies developing AI software tools for medical use are facing the risk of shutting down. In other words, AI software tools for medical applications that have been developed so far are not being consumed in real-world clinical practice. Why?

The most important criterion for adopting AI technology in medical practice is that the technology should help provide better quality care for patients and improve healthcare outcomes of the patients, that is, create quality and value for patients. One notable example of failure by not fulfilling this requirement is IBM’s Watson for Oncology. This AI software designed to provide information to assist cancer diagnosis was released in 2013 by IBM and was introduced by 8 hospitals in Korea (Republic of) in 2016 and 2017 (none since 2018) [4]. However, unlike what has been exposed to the public, diagnostic suggestions provided by Watson for Oncology were not as accurate as expected. As a result, the initial atmosphere of triumph was soon superseded by a sober evaluation of advantages and drawbacks [5,6]. In fact, the MD Anderson Cancer Center, one of the best cancer hospitals in the United States, attempted to introduce Watson for Oncology early in 2017, but found problems and stopped the project after having spent $62 million [7]. Also, IBM laid off approximately up to 70% of staff in the corresponding business division in the first half of 2018 [8]. Just as drugs and any other medical devices are required to pass a strict validation of safety and efficacy before they can be used for patients, thorough clinical validation before clinical adoption is critical for AI technology, too, regarding how accurate it is and how large of a benefit with patient care it can provide without creating any inadvertent harms [9-13].

One of the key points to consider when evaluating the accuracy of an AI algorithm is external validation, i.e., testing the algorithm accuracy using datasets collected independently from the training dataset [11,14-17]. This is due to strong data dependency of AI algorithms. The process of training AI algorithms is different from that of human learning, as the latter is based on understanding concepts and principles, while the former is based on the search for patterns in given data without an understanding of concepts and principles [18]. When a large amount of data is input to a computer, it creates mathematical formulae (i.e., a mathematical representation of the patterns) that associate the data to answers to afford AI. Modern AI technologies such as deep learning are known to have high accuracy compared to past technologies in finding the patterns. However, they have a strong dependency on training data. While high accuracy is generally guaranteed within the data used for training, the accuracy for data that were not used for training can be low. The accuracy of AI algorithms cannot go beyond the information inherent to the datasets on which they are trained and cannot avoid the biases and errors in the training data. This strong data dependency of AI poses a particular concern in the medical field [19]. The datasets used to train AI algorithms for medical applications are prone to various selection biases and may not adequately represent target populations in real-world clinical practice for many reasons [19]. Also, unexpected situations can occur in real-world clinical practice at any time, not infrequently [19]. As a result, there is a genuine risk that the accuracy of an AI algorithm may drop if the AI software is applied to the data and patients of another hospital or data acquired from other imaging systems or methods [13,20-23]. Likewise, it is uncertain how accurately an AI algorithm would perform in various real-world practice settings until it is validated directly in such clinical environments.

For proper validation of the clinical accuracy of AI algorithms, the test data should have the following features [11,16,17]: (1) data collected avoiding biases from specific indications (i.e., target patient groups in well-defined clinical scenarios) to which the AI software will be applied; (2) data from hospitals other than the institution in which the data for AI training were collected, and (3) data collected from multiple institutions. Also, whenever possible, prospectively collected data should be used [11,14,16,17]. However, most AI software applications for medical use developed until now have not been validated in this way [24], and the lack of appropriate clinical validation for AI algorithms, a phenomenon referred to as ‘digital exceptionalism,’ raises a significant concern [25,26].

The second criterion for adopting AI technology in medicine is that it should support healthcare providers or hospital administration if not directly helps patients. One successful example well addressing this point is an AI software developed and introduced by the University of Pittsburgh Medical Center (UPMC) [27]. This software analyzes data of hospitalized patients and estimates the probability of re-hospitalization within seven and 30 days after discharge. In the United States, the hospital may not receive reimbursement from insurers if a patient is re-hospitalized within a short period after discharge, and the early re-hospitalizations may ultimately work as a disadvantage to the hospital when it makes a contract with insurers. Therefore, it is crucial for hospitals to reduce the rate of early re-hospitalizations. UPMC is now reported to be reaping the benefits of its investment of $100 million in the enterprise analytics [27].

## What can artificial intelligence do for medicine?

If properly designed and used, AI technology could reinforce many weaknesses in current medical practice [1]. If time-consuming processes that require simple repetitive work are taken care of by AI, it would substantially reduce the fatigue of healthcare providers, and physicians could spend more time in facing with patients and concentrating on more complicated medical tasks [1]. AI technology may also reduce the number of inadvertent errors in clinical practice and may decrease differences in judgments among medical professionals. If patient conditions can be monitored 24 hours a day by AI systems, which would practically be impossible for humans to do, the patients may be managed more safely. Furthermore, new patterns discovered by AI through the analysis of big data from clinical practice may lead to the development of new biomarkers for diagnosis and treatment. Inputs by medical professionals who understand medicine, specific details of clinical practice, as well as patients, are critical for realizing these expectations. Many medical AI software applications developed to date were created mainly to make use of large data that happen to have already been available rather than addressing the actual needs identified by practitioners in real clinical world (i.e., definition of problems or pertinent use cases in real-world practice first, followed by data collection to address the needs). It is another reason why AI software applications developed so far are rarely used in clinical practice and highlights the importance of input by medical professionals who actually take care of patients on the spot.

## Which medical sector will be most affected by artificial intelligence?

It was spoken merely a few years ago that AI might soon replace specialists in radiology or pathology departments. However, with increasing understanding of AI, we now know that these premature “predictions” are only revealing a very shallow understanding of the technology and its application in medicine at the time. Do we not have to worry about AI replacing physicians then? In many cases, AI tools for medicine mostly play the role of a virtual assistant for physicians and healthcare systems, helping them to provide more accurate and efficient patient care, of which typical examples are many AI tools that have been being developed in the fields of radiology and pathology. On the other hand, in the setting of managing common chronic illnesses or primary healthcare, AI could be designed and used as a virtual assistant for patients and the public [1]. For example, patients pondering about whether or not to visit physicians for counseling or examination related to their minor health issues or patients wanting their prescription for chronic medication reissued might want to have AI do some of these functions so that they could save hospital visits. A similar scenario may also apply to emergency room visits. For example, if an AI system can make a suggestion using skin photographs taken with a smartphone in a child who has skin rash and fever at night regarding whether the child should visit the emergency room immediately or visit a pediatrician’s office the following day, the number of children visiting the emergency room during the night might decrease. In these scenarios, AI algorithms provide information directly to the patients and enable them to take their healthcare into their own hands. The work for developing AI algorithms of this kind has lagged behind AI for clinicians and healthcare systems [1]. However, AI tools for directly coaching patients and the public about common chronic conditions and mild health issues will likely soon become a major topic for discussion regarding AI in medicine given the large volume that these take in healthcare. Medical professionals in the AI era have an important responsibility in clinically validating the tools, providing trustworthy information about them, and making the right decisions about their adoption in the best interest of the patients.

## What should medical students do to prepare themselves for artificial intelligence?

Medical students should acquire the appropriate knowledge and experience required for them to act as ones who take the ultimate responsibility for their patients when applying the AI technology to them. Preparing for AI does not merely mean learning information technology such as computer programming. One should acquire sufficient knowledge of basic and clinical medicines (which constitute the fundamentals of medical practice and are keys to understanding how to use AI for medicine), data science, biostatistics, and evidence-based medicine. Even as a medical student, one should not passively accept stories related to AI in medicine in the media and on the Internet. Medical students should try to develop abilities to distinguish correct information from hype and spin [28] and even capabilities to create thoroughly validated, trustworthy information for patients and the public. While the curricula at medical schools would have yet to evolve to accommodate the educational needs sufficiently, some medical colleges in Korea (Republic of) such as University of Ulsan and Yonsei University have recently started providing AI-dedicated elective courses to the students.

## Conclusion

AI is expected to affect various fields of medicine substantially and, if properly designed and used, has the potential to reinforce many weaknesses in current medical practice and improve many aspects of healthcare. Healthcare professionals are responsible for ensuring that AI becomes a technology beneficial for patient care. Medical students should develop abilities to distinguish correct information about AI from hype and spin and even capabilities to create thoroughly validated, trustworthy information for patients and the public to prepare for the era of AI in medicine.

## Supplementary materials

Supplement 1. Audio recording of the abstract.
